# Use of Machine Learning and Statistical Algorithms to Predict Hospital Length of Stay Following Colorectal Cancer Resection: A South African Pilot Study

**DOI:** 10.3389/fonc.2021.644045

**Published:** 2021-10-01

**Authors:** Okechinyere J. Achilonu, June Fabian, Brendan Bebington, Elvira Singh, Gideon Nimako, Rene M. J. C. Eijkemans, Eustasius Musenge

**Affiliations:** ^1^ Division of Epidemiology and Biostatistics, School of Public Health, Faculty of Health Sciences, University of the Witwatersrand, Johannesburg, South Africa; ^2^ Medical Research Council/Wits University Rural Public Health and Health Transitions Research Unit (Agincourt), School of Public Health, Faculty of Health Sciences, University of Witwatersrand, Johannesburg, South Africa; ^3^ Wits Donald Gordon Medical Centre, School of Clinical Medicine, Faculty of Health Sciences, University of Witwatersrand, Johannesburg, South Africa; ^4^ Department of Surgery, Faculty of Health Science, University of the Witwatersrand Faculty of Science, Parktown, Johannesburg, South Africa; ^5^ National Cancer Registry, National Health Laboratory Service, Johannesburg, South Africa; ^6^ Industrialization, Science, Technology and Innovation Hub, African Union Development Agency (AUDA-NEPAD), Johannesburg, South Africa; ^7^ Julius Center for Health Sciences and Primary Care, University Medical Center, Utrecht University, Utrecht, Netherlands

**Keywords:** colorectal cancer, hospital length of stay, risk factors, support vector machine, logistic regression, internal validation techniques, prediction models, South Africa

## Abstract

The aim of this pilot study was to develop logistic regression (LR) and support vector machine (SVM) models that differentiate low from high risk for prolonged hospital length of stay (LOS) in a South African cohort of 383 colorectal cancer patients who underwent surgical resection with curative intent. Additionally, the impact of 10-fold cross-validation (CV), Monte Carlo CV, and bootstrap internal validation methods on the performance of the two models was evaluated. The median LOS was 9 days, and prolonged LOS was defined as greater than 9 days post-operation. Preoperative factors associated with prolonged LOS were a prior history of hypertension and an Eastern Cooperative Oncology Group score between 2 and 4. Postoperative factors related to prolonged LOS were the need for a stoma as part of the surgical procedure and the development of post-surgical complications. The risk of prolonged LOS was higher in male patients and in any patient with lower preoperative hemoglobin. The highest area under the receiving operating characteristics (AU-ROC) was achieved using LR of 0.823 (CI = 0.798–0.849) and SVM of 0.821 (CI = 0.776–0.825), with each model using the Monte Carlo CV method for internal validation. However, bootstrapping resulted in models with slightly lower variability. We found no significant difference between the models across the three internal validation methods. The LR and SVM algorithms used in this study required incorporating important features for optimal hospital LOS predictions. The factors identified in this study, especially postoperative complications, can be employed as a simple and quick test clinicians may flag a patient at risk of prolonged LOS.

## 1 Introduction

Surgical resection remains the principal treatment modality for patients with colorectal cancer (CRC), and the primary aim is to cure the disease (1–3). However, the economic burden of CRC treatment from presentation to post-surgery supportive care is high, requiring more cost-effective management plans that will benefit the patient or the healthcare providers (1, 4). South Africa has a two-tiered healthcare system that includes a national health insurance system servicing approximately 20% of the population and a state health system servicing the remaining majority of the population (5). Irrespective of the health sector to which patients present for care, there is a need to develop prediction models that might identify those at increased risk of prolonged hospitalization during their treatment for CRC. Length of stay (LOS) is an easily accessible indicator to measure resource utilization, which speaks to performance and efficiency. A prolonged LOS impacts resource allocation and has been associated with increased risk of several postoperative complications, contracting hospital infections, and hospital readmission (6, 7). An accurate prognosis prediction of LOS is desirable for healthcare management, hospital resource utilization, successful treatment, and discharge planning, especially in low- to middle-income countries such as South Africa. Once a prognosis model is established, efforts can be directed toward identifying risk factors to reduce hospital LOS.

Hospital LOS greater than the mean or median has been used to define prolonged LOS (6, 8–10). Due to variations in patient care and management or response to treatment, the median as a central tendency is consistently and considerably used as a better indicator of LOS than the mean. In any of these measures, several predictors of LOS have been identified, which vary across studies. Factors such as patient age at diagnosis and surgical complications have been consistently recognized in most studies. There is an increase in the use of traditional statistical approaches, such as logistic regression model in predicting LOS (4, 6–9). However, in a multifactorial prediction, detecting interactions and assessing the combination of statistically significant predictors may be challenging with standard statistical procedures. Studies have reported that a more reliable and improved prognosis prediction is achievable using machine learning (ML) and artificial intelligence approaches (11, 12).

Francis et al. (10) investigated the use of a multilayered perceptron neural network (MLPNN) to predict delayed discharge and readmission after CRC surgical resection. The dataset consists of 275 patients who were scheduled for laparoscopic surgery between 2002 and 2009. A median LOS greater than 6 days was used to define prolonged hospital LOS (10). The MLPNN model achieved an area under the receiving operating characteristics (AU-ROC) of 0.817, which was slightly higher when compared with that of logistic regression (AU-ROC = 0.807) using a split-sample method. Independent validation with an insufficient sample size has been shown to be misleading in many studies (13, 14). The study of Francis et al. (10) failed to report the confidence interval of the AU-ROC estimates, which made it difficult to measure the uncertainties in the performance estimates of the model. A study by Stoean et al. (15) estimated LOS using the ensemble of support vector machine (SVM), neural network, logistic regression (LR), and decision tree algorithms. A total of 368 patients were analyzed, and the length of stay was divided into three categories. Using random cross-validation (CV) with 30 repeats, the authors showed the highest accuracy of 73.14 ± 4.37, achieved by the ensemble approach.

Internal validation refers to a validation based on the test data from a similar population (16). Internal validation methods such as CV and bootstrap aim to provide more accurate estimates of the performance of a predictive model as compared to the split-sample method (13). CV is a sophisticated resampling approach and has become the standard procedure in estimating the internal validity of a predictive model. However, studies have shown that, in some settings, the bootstrap method outperforms CV (13, 17). With this in mind, we differentiated our study from previous studies by comparing the efficiency of the repeated 10-fold CV, Monte Carlo CV, and bootstrap (0.632 method with replacement) methods for predictive SVM and LR models. Our overall aim was to develop classifiers to distinguish short from prolonged hospital LOS and identify previously unrecognized features that influence hospital LOS. Prediction models for hospital LOS for CRC patients undergoing surgery in South Africa have not been developed. Such models can contribute valuable information to healthcare providers that would, ideally, enhance the care of affected patients and improve the efficiency of healthcare provision.

## 2 Material and Methods

### 2.1 Study Data

The dataset was extracted from the 2015–2019 CRC in South Africa (CRCSA) study, a multi-ethnic urban cohort study conducted in Johannesburg, South Africa. The CRCSA study aimed to improve local statistical reporting and the clinical management of patients with CRC. In total, 716 adult patients were recruited during the study period. The methodology of the CRCSA study has been detailed in a prior publication (18). Of the total sample (*n* = 716), we extracted 383 patients undergoing surgical resection with curative intent, irrespective of whether the surgical method was laparoscopic or open. These patients underwent different surgical procedures and were grouped into segmental colectomies, major resections, and others, which was included as a variable in the predictive analysis. Palliative surgery and surgery for local and distant metastatic disease were not considered in this study. Ethical approval for this study was obtained from the Human Research Ethics Committee (Medical) of the University of the Witwatersrand, Johannesburg, South Africa (M1911131).

Four hospitals from the University of Witwatersrand Academic Teaching Hospital complex were included in the CRCSA study, namely, Wits Donald Gordon Medical Centre (WDGMC), a private academic teaching hospital, Charlotte Maxeke Johannesburg Academic Hospital (CMJAH), Chris Hani Baragwanath Academic Hospital (CHBAH), and Edenvale Hospital. Of these, the former three hospitals function as tertiary referral centers, while the latter, Edenvale Hospital, functions as a secondary treatment center. All the patients in public hospitals were grouped as a new variable “hospital” with two categories, “public” and “private.” Patients treated at WDGMC were categorized as “private,” and those receiving care at CMJAH, CHBAH, and Edenvale Hospitals were categorized as “public.” Some of the patients, especially in the public hospitals, experienced longer waiting times for surgery after hospital admission. Hence, the primary outcome variable (LOS) was based on the number of days spent in the hospital following surgery. LOS was defined in days as the interval between the day of surgery and the day of hospital discharge. A prolonged hospital LOS was defined as LOS that exceeded 9 days, which is the median LOS in the CRC study. Clinical data captured in the CRCSA study were based on the literature and clinical domain knowledge. The clinical information included socio-economic and demographic characteristics, family history, laboratory and clinical testing, and medical and surgical histories (**Table 1**). Data pre-processing included feature engineering and imputation of missing values.

**Table 1:** Features assessed in the prediction models.

Category	No. of features	Description
Demographics and socioeconomic	12	Age at time of first visit, gender, race, language group, place of birth
		Province, travel distance, relationship status, employment
		Education, family history of cancer, relationship to patient
Medical and surgical history	22	Referral, smoking, alcohol consumption, diabetes mellitus, hypertension
		Gastrointestinal symptoms, non-cancer therapies, previous cancer diagnosis, pre-therapeutics
		Weight, height, BMI, malignancy location, radiological stage, treatment decision
		Colonoscopy, ECOG performance status, anesthetic grading assessment, hemoglobin
		Radiation complication, chemotherapy complication, chemotherapy treatment
Histology	10	Histological evidence of colorectal cancer, excision margin, grade of differentiation
		Subjective grading, histology, total number of lymph nodes, number of positive lymph nodes
		T stage, M stage, N stage
Surgical procedure	18	Complications, pre-therapeutic surgery, enterostomal therapist seen
		Dietician seen preoperatively, surgical urgency, treatment intent, time to surgery
		Surgeon performing operation, treatment intent, surgeon, pre-stoma type
		Surgical access, cancer complications at transplant, anastomosis, laparoscopic complications
		Stoma, postoperative complications, anastomotic technique, anastomotic type

**Figure 1:**
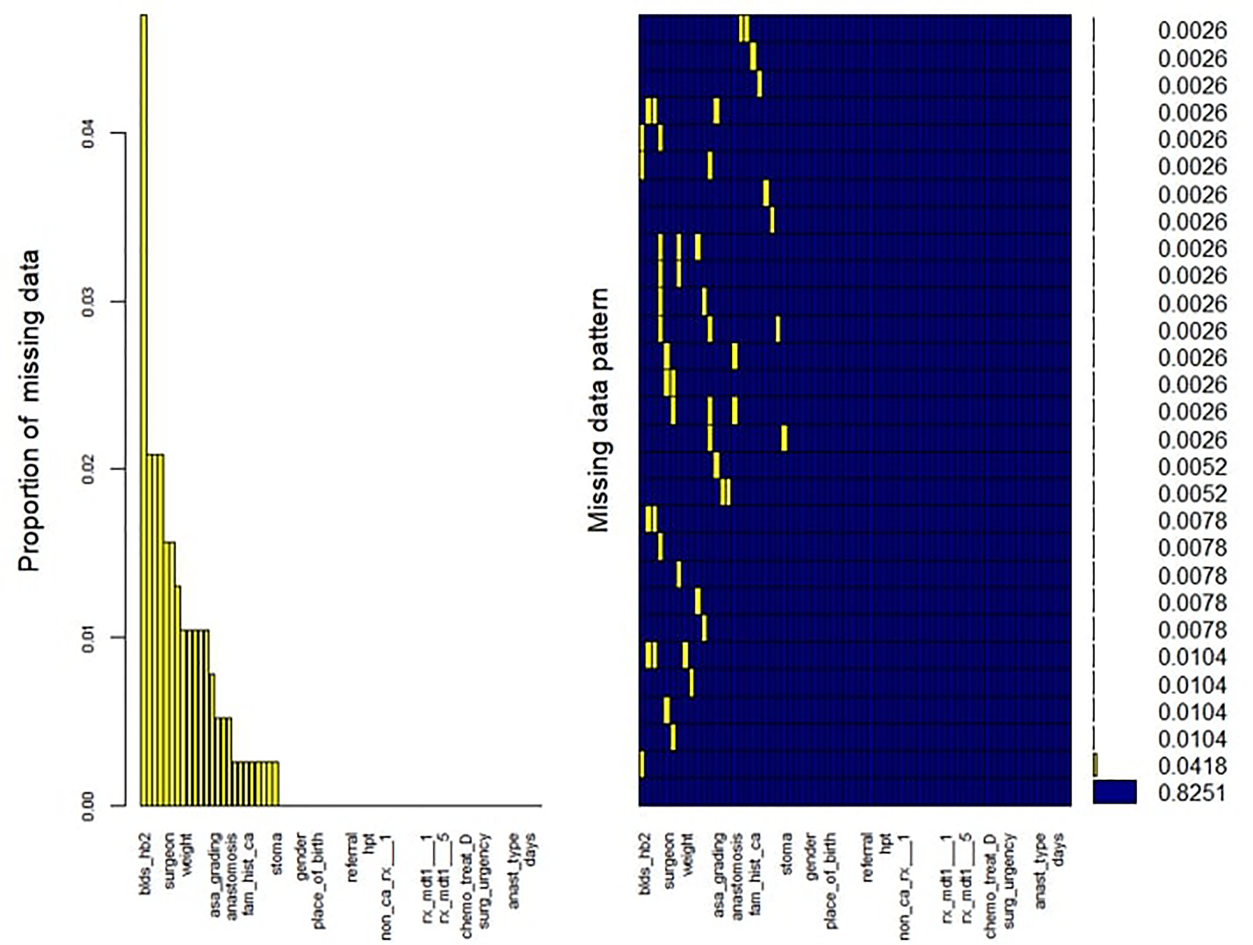
Missing data pattern and proportion in the CRCSA study.

Overall, 83% of CRC patients on the CRCSA database had completed records with no missing information (**Figure 1**). For those patients with incomplete observations, the missingness within each variable was not related to its value or any other variable in the database. We identified 25 out of 69 variables with one or more missing value(s). The proportion of missing values for each variable with missing records was computed. The variable pre-surgical hemoglobin (Hb) had the highest proportion of missing values, with about 5% missingness. Little’s missing completely at random (MCAR) test (19) demonstrated that missingness was completely at random (*p* = 0.304) (**Figure 1**). The MissForest imputation method (20) was used to replace missing values. MissForest is a non-parametric method of imputation based on the random forest algorithm (21). The out-of-bag errors estimated by the MissForest method were 0.04 for the continuous variables and 0.16 for the categorical variables imputed in this study, thus validating the reliability of the MissForest method.

### 2.2 Choice of Classifier

#### 2.2.1 Logistic Regression

We considered a classification problem of separating a set of training samples belonging to two classes: in this study context, short LOS or prolonged LOS.


(1) $$(x_{i},y_{i}),x_{i}\in R^{n},y_{i}\in \{-1,+1\},i=1,2,\ldots ,n$$

Where *x_i_* is an *n*-dimensional real valued features that belong to either one of the two classes (*y_i_* ∈{–1, + 1}). The objective is to define a function [*f(x)* = *y*] that can correctly classify patients into one of the two classes based on the feature vector. LR is a statistical technique in which the response variable (*y*) has a binomial distribution (10, 15). Given a set of features *x_i_*, LR regression determines the membership probability for one of the two classes using


(2) $$P=\frac{e^{\beta +\beta_{i}x_{i}}}{1+e^{\beta +\beta_{i}x_{i}}},$$

where *β* = (*β*_1_, *β*_2_, …, *β_m_*) ∈*R^m^* and are determined by maximum-likelihood estimation. LR has gained popularity in predicting hospital LOS.

#### 2.2.2 Support Vector Machine

SVM is a machine learning algorithm introduced by Vapnik (22). Its application has been promoted in different studies due to its capacity to perform classification and regression based on statistical learning theory and structural risk minimization. Also, it has the ability to handle high-dimensional datasets and linear and nonlinear problems with high performance accuracy (15). Considering the example in Equation 1, where the classes are linearly separable, SVM finds a maximum or optimal hyperplane that gives the greatest separation between the positive and the negative classes (between short LOS and prolonged LOS). A separating hyperplane is defined as *w.x* + *b* = 0, where *w* is the weight vector, which denotes the orientation of the hyperplane, and *b* denotes bias term. A set of hyperplane margins (||*w*||^–1^) are maximized based on a Lagrangian multiplier (*α_i_*) to identify the optimal margin. The classifier function can be expressed as:


(3) $$f(x)=\text{sgn}\left(\sum_{i}^{n}y_{i},\alpha_{i}x.x_{i}+b\right)$$

for data that are linearly separable. In this study, we identified that the nonlinear (radial basis function) variant of SVM was more appropriate. In this case, the input features are mapped into a high-dimensional space and the optimal margin is constructed using the radial kernel function, *k*(*x*_1_, *x_j_*) = exp(–||*x_i_* – *x_j_* ||^2^/2σ^2^). Hence, the classifier function can be expressed as:


(4) $$f(x)=\text{sgn}\left(\sum_{i}^{n}y_{i},\alpha_{i}kx.x_{i}+b\right)$$

### 2.3 Model Development and Validation for Prognosis Prediction

We developed predictive models using LR and SVM (with radial basis kernel) with the “caret package” implemented in the R software. The least absolute shrinkage and selection operator (LASSO) was used to select the most informative features (23). LASSO, which is a penalized regression method, greatly depends on the choice of the tuning parameter (λ) to select the optimal model. The LASSO regression formulation is defined as:


(5) $${\text{minimize}}_{\beta }\sum_{i=1}^{n}{\left(y_{i}-\beta_{0}-\sum_{j=1}^{p}B_{j}X_{ij}\right)}^{2}+\lambda \sum_{j=1}^{p}\left|\beta_{j}\right|$$

where β denotes the regression coefficients and λ the tuning parameter. The objective of tuning a ML hyperparameter is to limit model overfitting because it would lead to poor performance on unseen data. The hyperparameter λ was tuned by using an internal 10-fold CV (repeated 30 times), Monte Carlo CV, and bootstrap (both of which were repeated 10 times). To optimize the choice of the hyperparameter, grid search over λ = {0.01,0.038 by 0.0001} was performed. The hyperparameter value resulting in the highest AU-ROC was selected as the best λ for the final model. Features with non-zero coefficients in the LASSO model were identified as the most relevant features and are used as input features for the LR and SVM models. In addition, the ranking of the feature importance according to each specified model was visualized. For the SVM classifier, the hyperparameters were tuned using the three validation methods specified above for the LASSO model. The values of the radial basis function kernel width (*σ*) and the loss penalty term (*C*) were searched over the ranges of *σ* = {0.0005,0.005,0.045,0.05,0.08,0.01,0.10,1.00} and *C* = {0.1,0.5,1.0,1.50,1.60,1.65,1.89,1.95,2.00}. Using the default parameters, we fitted the LR models with the three validation methods, as stated previously. The average fit of each classifier per model fitted was used to determine its performance. For the two classifiers used in this study (LR and SVM), a total of six models were developed based on the three internal validation methods.

### 2.4 Evaluation

The ROC and calibration plots were used to evaluate the performance of the LR and SVM models across the different resampling methods. These methods offer ways to visualize the quality of a prediction model (24). ROC is used to characterize the performance of a predictive model across a set of possible thresholds between the sensitivity and specificity of the model (24). The probability values of a classifier are retrieved, and a point is specified. Values higher than the threshold are classified as positive (prolonged LOS); otherwise, they are classified as negative (short LOS). The higher the performance, the better the algorithm discriminates patients into the outcome classes. A perfect classifier would yield a point at the 0,1 of the ROC space (AU-ROC = 1.0), while a line of no discrimination would yield points at the diagonal. A calibration plot is a line plot that shows the agreement between the actual outcome and the predicted outcome given by the model. For instance, if the LR model predicts a 40% risk of a prolonged hospital LOS for a patient in this study, it is expected that approximately 40 out of 100 patients with such prediction should have the observed frequency of prolonged LOS. A perfect calibrated plot should have a line along the 45 line; hence, the closer the points to the diagonal, the more reliable are the model predictions. The evaluation plots were estimated using the pROC, caret, and ggplots packages in R software. The Wilcoxon signed-rank test was used to conduct a pairwise comparison of the LR and SVM models to examine whether the differences in the estimates of AU-ROC are statistically significant. Two-sided tests were employed, and a *p*-value <0.05 was considered statistically significant. This non-parametric test has been used in several studies for the comparison of predictive models (25). We also compared the sensitivity and specificity of the models to evaluate the model performance further. The R source codes for this study have been deposited in the Github platform to reproduce the study models (https://github.com/KechJay/HLOS_LS).

## 3 Results

**Figure 2:**
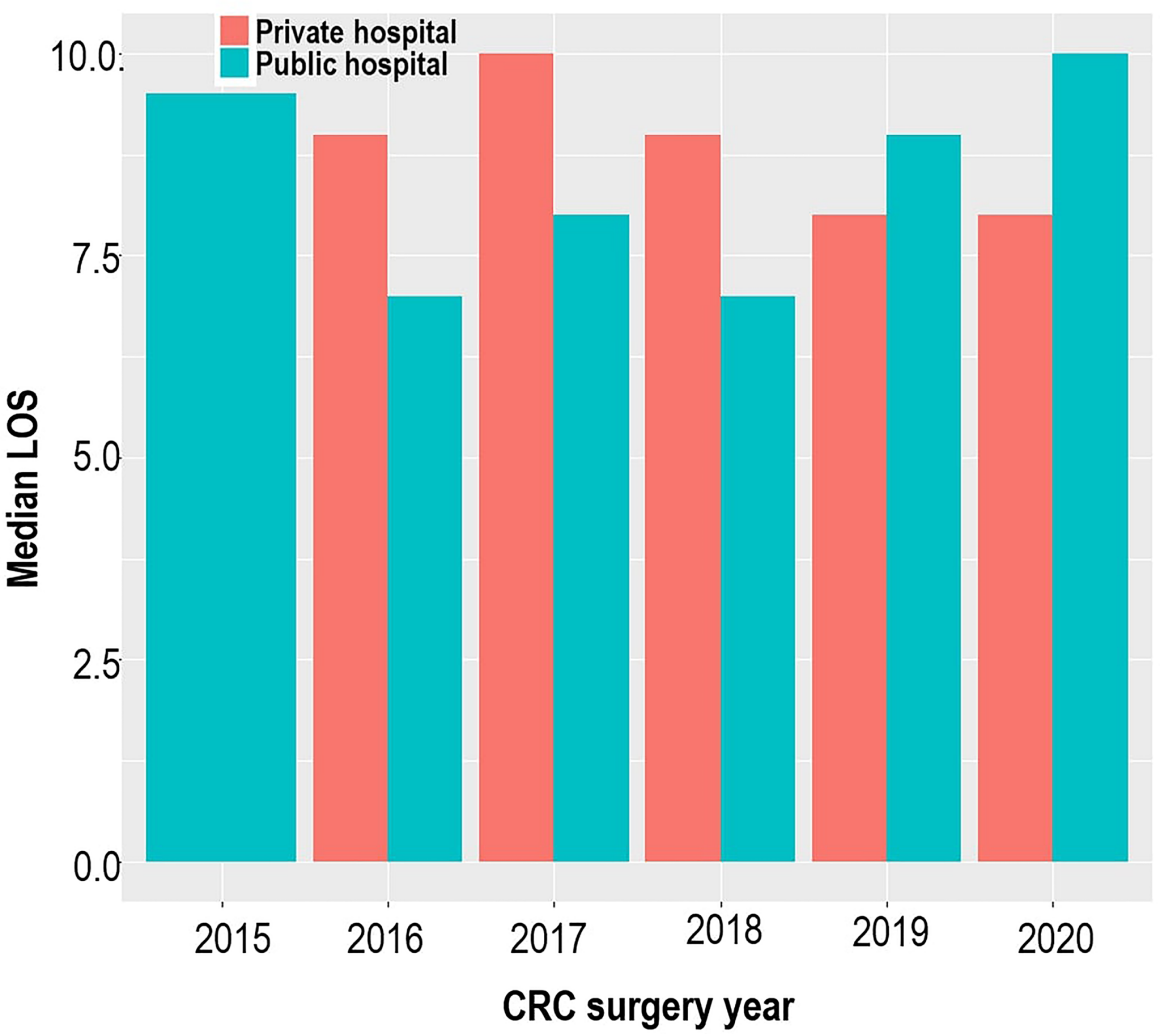
Bar plot illustrating the median hospital length of stay across the study period and study sites.

A total of 383 patients who underwent CRC resection between 2015 and 2020 were studied. Of these, 53.5% had an open surgical procedure, 38.1% had a complete laparoscopic procedure, and 8.4% had a laparoscopic-assisted procedure or a laparoscopic procedure that was converted to an open procedure. The mean age of the 383 patients was 58 (±12.9) years, with equal proportions of men and women. Most patients were of self-reported white (46.9%) and black (36.8%) ethnicity. In total, 202 (52.7%) patients were treated in a private facility and 181 (47.3%) patients were treated in public facilities. **Figure 2** shows no consistent pattern in the distribution of the median LOS for private and public hospitals across the study period, and there was no significant difference in LOS when comparing private and public facilities (OR = 0.77, *p* = 0.214). Overall, when the admissions were pooled over the study period, the median LOS was 9 days, with 53% of patients spending more than 9 days in the hospital. The median LOS values for open and non-open surgical procedures were the same (9 days), 9 days for elective surgery, and 8 days for non-elective surgery.

**Figure 3:**
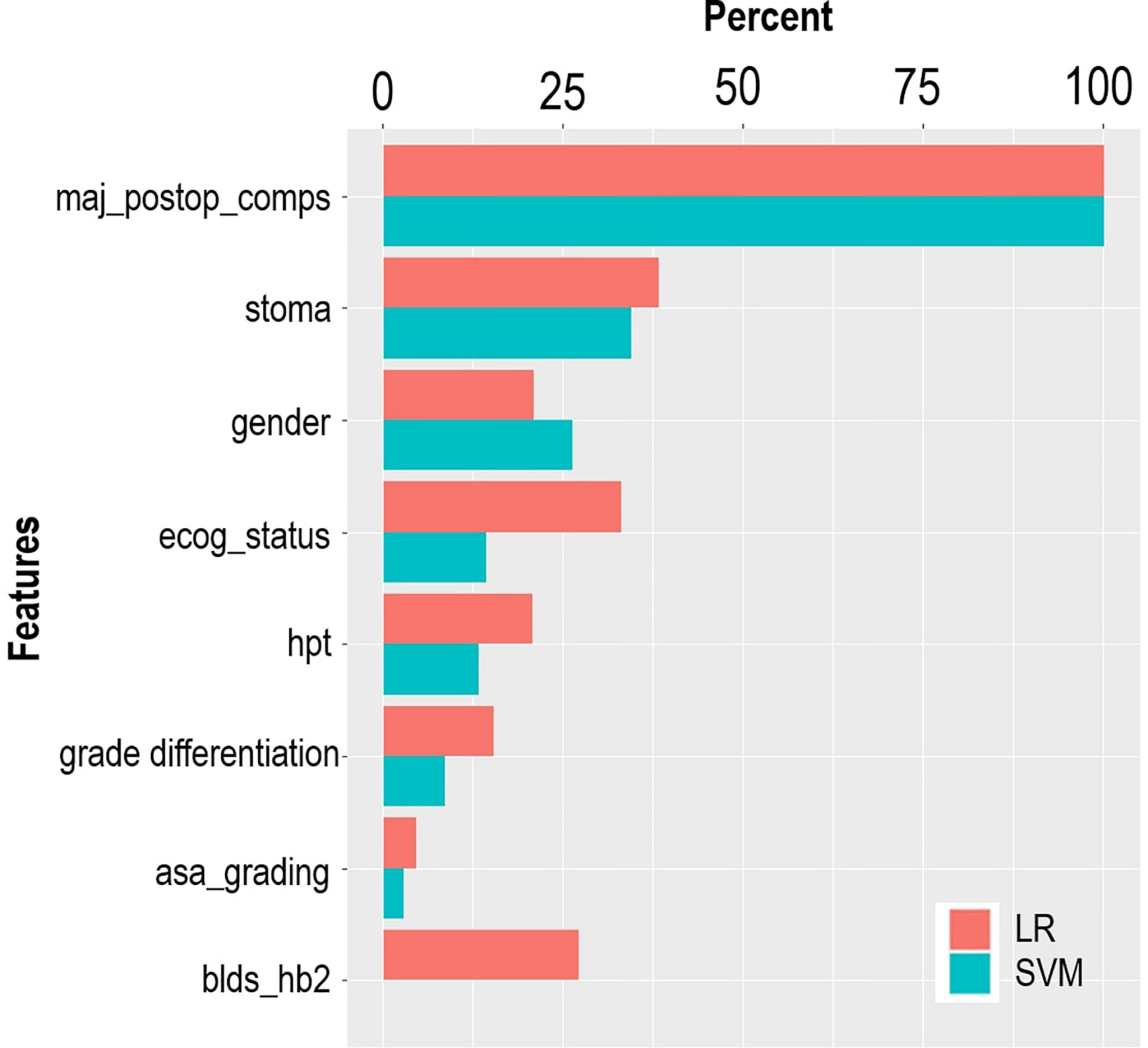
Variable importance ranked by LR and SVM.

The LASSO feature selection method identified a set of eight features, which were consistent across the three validation methods. The selected features were used as input to the LR and SVM models. **Table 2** provides the distribution of the input features across the outcome variable (hospital LOS). According to the LR model, most of the predictive features are significantly associated with the risk of prolonged LOS at a 5% significant level (**Table 2**). The risk of prolonged LOS is reduced with female patients and patients with increased hemoglobin levels (measured preoperatively). Other factors, such as having a postoperative complication, stoma, and patients presenting with a history of hypertension, increased the risk of prolonged LOS. For instance, the results showed that a postoperative complication was a major significant risk of a protracted hospital LOS. Of the patients with prolonged LOS, 69% had postoperative complications compared with 31.1% of patients without postoperative complications. Results from the LR model showed that patients with postoperative complications had approximately 14 times the odds of experiencing prolonged LOS compared with patients without postoperative complications. The relevance of these features is depicted in **Figure 3**. According to LR and SVM, a postoperative complication was the most relevant feature in this study. Most of the features were ranked higher in LR compared to that in SVM. Tumor grade differentiation and anesthetic grading assessment are the least ranked features based on the two models.

**Table 2:** Factors predicting prolonged length of stay (LOS) in colorectal cancer (CRC) patients after surgical resection.

Characteristics	Short (≤9 days)	Long (>9 days)	Total	OR (95%CI)
N	203	180	383	
Hemaglobin (blds_hb2)	12.0 ± 2.7	12.0 ± 2.9	12.0 ± 2.8 g/dl	0.89 (0.81–0.98)
Postoperative complications (maj_postop_comps)				
No	164 (80.8)	56 (31.1)	220 (57.4)	1.00
Yes	39 (19.2)	124 (68.9)	163 (42.6)	13.56 (7.76–23.69)
ECOG performance status (ecog_status)				
ECOG0	52 (25.6)	32 (17.8)	84 (21.9)	1.00
ECOG1	75 (36.9)	66 (36.7)	141 (36.8)	1.74 (0.87–3.50)
ECOG234	40 (19.7)	57 (31.7)	97 (25.3)	3.37 (1.59–7.15)
Test not done	36 (17.7)	25 (13.9)	61 (15.9)	1.01 (0.43–2.39)
Stoma				
No	96 (47.3)	53 (29.4)	149 (38.9)	1.00
Yes	107 (52.7)	127 (70.6)	234 (61.1)	2.54 (1.47–4.37)
Gender				
Male	88 (43.3)	103 (57.2)	191 (49.9)	1.00
Female	115 (56.7)	77 (42.8)	192 (50.1)	0.55 (0.33–0.92)
History of hypertension (hpt)				
No	136 (67.0)	107 (59.4)	243 (63.4)	1.00
Yes	67 (33.0)	73 (40.6)	140 (36.6)	1.76 (1.03–3.00)
ASA grading assessment (Asa_grading)				
Grade I	35 (17.2)	25 (13.9)	60 (15.7)	1.00
Grades II–III	75 (36.9)	82 (45.6)	157 (41.0)	1.30 (0.61–2.80)
Test not done	93 (45.8)	73 (40.6)	166 (43.3)	0.92 (0.43–1.97)
Grade of differentiation				
Unknown	32 (15.8)	32(17.8)	64 (16.7)	1.00
Grade 2	11 (5.4)	12(6.7)	23 (6.0)	1.32 (0.40–4.32)
Grade 3	150 (73.9)	124(68.9)	274 (71.5)	0.70 (0.35–1.39)
Grade 4	10 (4.9)	12(6.7)	22 (5.7)	2.06 (0.63–6.77)

OR, odds ratio from the LR model; ECOG, Eastern Cooperative Oncology Group; ASA, American Society of Anesthesiologists.

**Figure 4:**
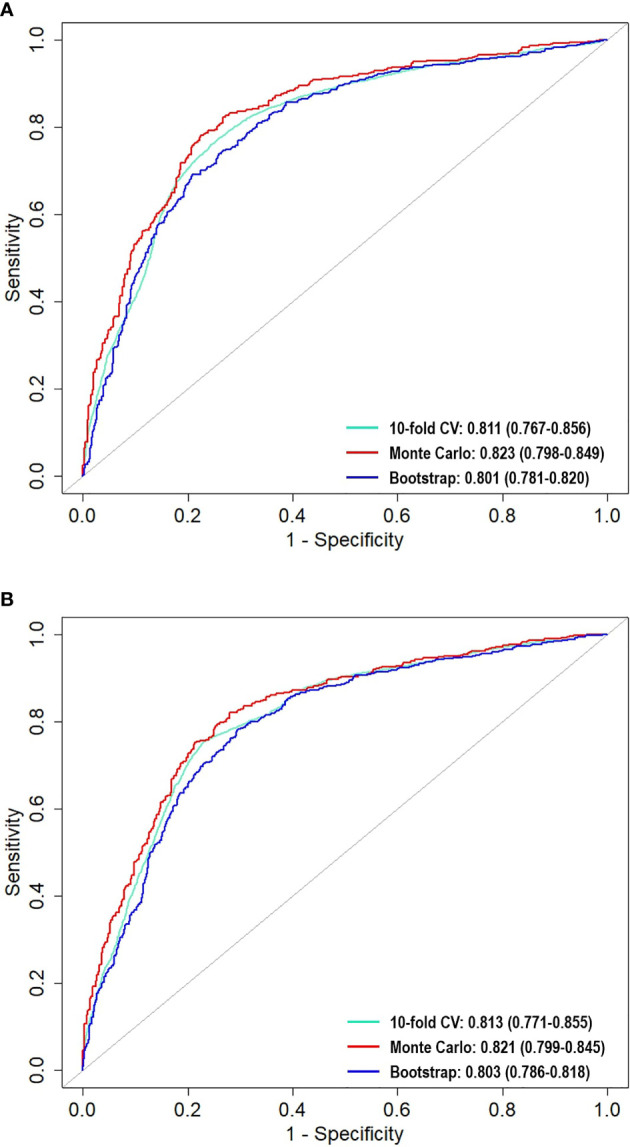
Comparison of area under the receiver operating characteristic curves (AU-ROC) across the resampling methods for **(A)** LR and **(B)** SVM.

The mean, standard deviation, and confidence intervals of the predictive models with the three validation methods are shown in **Table 3**. We also generated the ROC curve to visualize the predictive performance of the LR and SVM models (**Figure 4**). In these figures, the Monte Carlo method appears to demonstrate a slightly higher performance than that of the other validation methods in each predictive model, with the AU-ROCs reaching 82.3% and 82.1% for LR and SVM, respectively. However, it is known that a narrow confidence interval gives a narrower uncertainty for the ROC estimate (a more precise estimate). The bootstrap method resulted in a narrower confidence interval compared to that of the other methods in the two models. Nonetheless, we cannot claim that any of the methods is preferred because, for each internal validation technique, the LR and SVM models showed similarities in performance, and these hold for all the summary statistics. As expected, the Wilcoxon signed-ranked test showed no significant difference between the models across the different internal validation methods. The SVM model with the bootstrap validation method had the highest sensitivity (81.8%), which indicates that 81.8% of the patients were correctly classified into the prolonged hospital LOS class. The SVM with the 10-fold CV showed the most heightened sensitivity of 72%, although with the highest standard error.

**Table 3:** Average prediction results and standard deviations obtained by the logistic regression (LR) and support vector machine (SVM) models.

Classifier	Validation method	ROC ± SD	Sensitivity ± SD	Specificity ± SD
LR	10-fold CV	0.811 ± 0.070	0.798 ± 0.086	0.718 ± 0.107
	Monte Carlo	0.823 ± 0.040	0.784 ± 0.056	0.711 ± 0.087
	Bootstrap	0.801 ± 0.031	0.799 ± 0.028	0.678 ± 0.065
SVM	10-fold CV	0.813 ± 0.066	0.782 ± 0.088	0.722 ± 0.110
	Monte Carlo	0.821 ± 0.039	0.812 ± 0.043	0.689 ± 0.082
	Bootstrap	0.803 ± 0.026	0.818 ± 0.034	0.655 ± 0.047

ROC, receiver operating characteristics.

**Figure 5:**
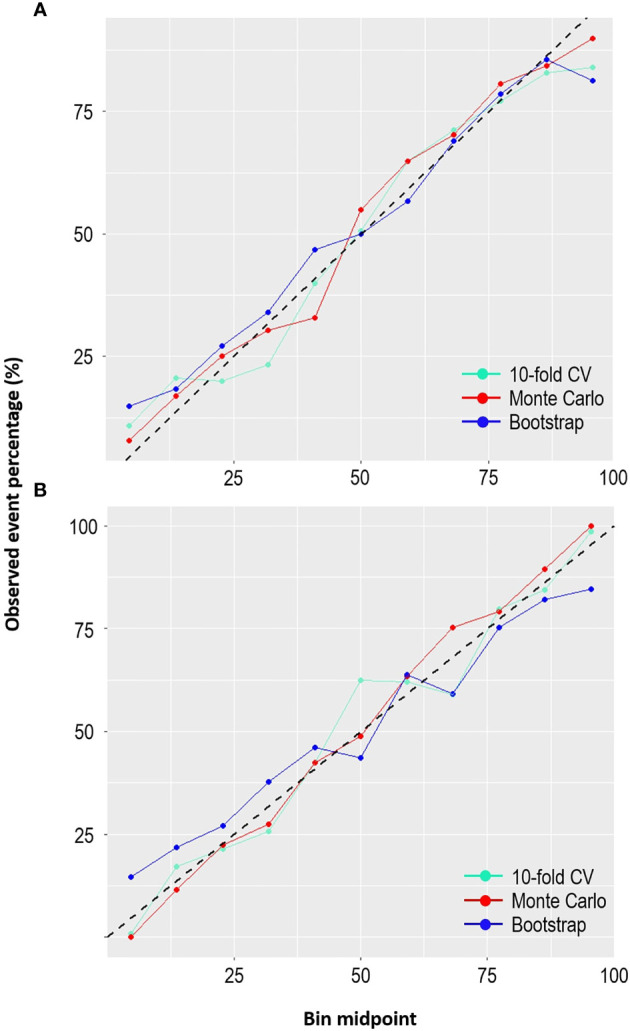
Comparison of the calibration plots across the resampling methods for **(A)** LR and **(B)** SVM.

Furthermore, the calibration plots (**Figures 5A, B**) demonstrated that the two models showed considerable agreement between the observed and predicted probabilities across the three internal validation methods. If the points are above the diagonal line, the predicted probabilities are minor; otherwise, they are too large compared to the observed probabilities. The LR model fitted with the bootstrap method appeared to have good calibration, except at the bottom left and right, where the model under- and over-predicted the probabilities. However, the SVM model with the Monte Carlo method seemed to be better calibrated than the SVM with other methods. Overall, the models showed little deviation, thus confirming good calibration.

A further analysis was conducted to determine factors that also predisposed patients to postoperative complications. Eight variables were identified by the LASSO method and were used to fit an LR model. The risk factors for postoperative complications are summarized in **Table 4**. Factors such as surgical type, the use of chemotherapy treatment, and having a pre-therapeutic or intraoperative complication increased the chances of a postoperative complication.

**Table 4:** Determinants of postoperative complications based on logistic regression.

Variables	No (221)	Yes (162)	OR (95%CI)
Procedure description			
Segmental colectomies	110	45	1.00
Major resections for rectal cancer	101	103	1.83 (1.07–3.16)
Combination/other	10	14	3.49 (1.33–9.15)
Chemotherapy treatment			
No	154	93	1.00
Yes	67	69	2.23 (1.37–3.62)
Hospital			
Private	103	99	1.00
Public	118	63	0.51 (0.31–0.84)
Pre-therapeutic surgical complications			
No	196	127	1.00
Yes	25	35	2.07 (1.08–3.95)
Smoking			
No	133	86	1.00
Yes	88	76	1.15 (0.72–1.83)
Perioperative complications			
No	186	127	1.00
Yes	35	35	1.86 (1.04–3.33)
Gender			
Male	101	90	1.00
Female	120	72	0.70 (0.44–1.10)
Primary tumor			
T123	22	12	1.00
T4	43	40	2.22 (0.91–5.43)
Tx	110	88	1.76 (0.76–4.05)
T0	46	22	0.96 (0.36–2.56)

## 4 Discussion

CRC is one of the most common cancers affecting South Africans, and models that improve the care delivered to patients and simultaneously enhance efficiency for service providers are desperately needed (15, 26). In this pilot study, the first of its kind for South Africa, we used machine learning models to determine the median LOS and investigated the factors impacting LOS for those with CRC undergoing surgical resection with curative intent.

Kelly et al. (6) noted that the median LOS values varied across studies and depend on the healthcare systems. We have acknowledged the longer median LOS observed in this study compared to other studies with shorter median LOS in higher-income countries. Lack of screening, the delay in diagnosis and treatment, inaccessibility to ICU bed postoperatively, and the inability to implement the enhanced recovery after surgery (ERAS) protocol may account for the longer median LOS observed in this study. Therefore, we assume that it is logical to extract the LOS from the study data because it is specific to the study population and speaks to the colorectal cancer healthcare in the studied region.

We investigated the ability of LR and SVM to predict hospital LOS and the effects of repeated 10-fold CV, Monte Carlo CV, and bootstrap internal validation methods on the models. Our study showed that these two models could effectively predict hospital LOS with high AU-ROC. Previous studies on LOS using machine learning procedures have shown that the accuracy in predicting prolonged LOS for patients undergoing CRC resection can be improved using ML procedures (10, 13, 14). In addition, ML encourages the reproducibility and generalizability of the developed model. It is noteworthy that the LR and SVM models reached predictive accuracy values of 0.823 and 0.821, respectively. These are slightly higher than that achieved by the MLPNN method used in Francis et al. (10). Our model accuracy measures (79% and 77%) for LR and SVM are higher than that achieved in the study by Stoean et al. (15). In the studies of Francis et al. (10) and Stoean et al. (15), other models were shown to outperform LR. Our study showed good performance with LR, even higher when compared with those of MLPNN and other models (results not shown). This indicates that performance may depend on the study data and the modeling procedure. We found no significant difference between the results of the two models used in this study; however, models with bootstrap followed by Monte Carlo CV methods resulted in minimum variability compared to those that used repeated 10-fold CV.

This study showed that the identified predictive risk of prolonged hospital LOS relates primarily to patient-related factors. Anemia in CRC increases blood transfusion risk during surgery and consequently prolongs hospital LOS (8, 27, 28). Preoperative hemoglobin was tested in the CRCSA study. Although this variable was modeled on a constant level, the relationship between this variable and those requiring blood transfusion at the time of surgery was unknown. However, the findings of this study correlated with those of previous studies (8, 27, 28). Several studies support the relationship between the requirement for a stoma and risk of prolonged hospital LOS (10, 27, 29). Furthermore, stoma type and length are also associated with prolonged hospital LOS (30). We found no significant impact of stoma type on prolonging LOS; however, having stoma formation compared to not having stoma formation increases the odds of extending LOS to about 2.5 times. As seen in other published studies, our study also confirms the significance of hypertension, Eastern Cooperative Oncology Group (ECOG) performance, the American Society of Anesthesiologists (ASA) grading assessment, and grade of differentiation on LOS after colorectal surgery (31–33).

Among the demographic features in this study, sex was the only one that significantly influenced prolonged LOS, and this was well described in other studies. The male patients in this study had a longer median LOS of 10 days than female patients who had a median LOS of 8 days. Hence, female patients had a 55% reduced odds of prolonged LOS compared to male patients. Male patients also showed the likelihood of increased postoperative complications. Previous studies have found that the rate of postoperative complications was significantly higher in male patients than that in female patients (34, 35). The feature selection method showed no evidence of a patient’s age being associated with prolonged LOS for patients undergoing CRC surgery. In a study done by Leung et al. (9), a patient’s age was shown not to have a significant impact on the hospital LOS. However, some studies have suggested that a patient’s age is one of the informative risk factors for prolonged LOS (6, 36). The median age of the patients in this study was 60 years (range = 18–91 years). Using this information, we further categorized the patients into age groups: <60 years (191 patients) and ≥60 years (192 patients). A comparison of the LOS between these age groups showed that both groups have a median LOS of 9 days. This further highlights the similarity in the LOS of these patients, irrespective of age.

A postoperative complication is a relatively fixed risk factor that influences LOS, which has been shown in previous studies (8, 9). This is a testament to the validity and reliability of both the study data and the modeling approaches used in the present study. In the ranking of variable importance in this study, postoperative complication was ranked 100% by LR and SVM, suggesting that it is a strong determinant of LOS. Most patients in this study had only one postoperative complication each. The majority of the postoperative complications experienced by the patients were ileus, surgical wound sepsis, anastomotic leak/breakdown, and access collection, with the first two being the most recorded complications. Also, the most common postoperative complication in this study, according to the Clavien–Dindo classification, is grade II (56%). A postoperative complication has also been identified as a factor that influences a patient’s overall survival (37).

It is essential to investigate further possible pre- or perioperative factors that predispose patients to postoperative complications. Our findings further showed that factors such as the type of procedure, pre-therapeutic and intraoperative complications, gender, preoperative chemotherapy, staging, and hospital category predispose a patient to postoperative complications. Previous studies supported this (34, 35, 38, 39). If these factors influencing postoperative complications in patients undergoing CRC surgery are identified and controlled, the impact of this variable on LOS may decline drastically, and the overall post-surgical quality of life of the patients may be improved.

Several strengths of the current study should be acknowledged. This was a population-based study, which includes patients diagnosed with CRC who underwent surgical resection within public and private hospitals in the Johannesburg region, linked to the Witwatersrand. These four hospitals have both private and public healthcare facilities, which serve the most extensive urban population in South Africa. There is a high level of confidence in the follow-up of the patients in this study, with a detailed collection of an array of local and established patient information. In addition, the model developed in this study could be extrapolated to other cancer centers across South Africa and may also apply to the general population of South Africa, given that the hospitals in this study are mostly referral hospitals. The study is also subject to a few limitations. There may have been surgical advances, treatment, and patient management across the study period that may have affected the hospital LOS patterns experienced by these patients. Also, inter-hospital variations may impact the hospital LOS because different hospitals may have disparities in hospital admission policies. In addition, the absence of the implementation of ERAS as a standard of care could be another limitation of this study.

Although the sample size in this study was small, and we agree it is a limitation, this is the first of such studies from South Africa and Sub-Saharan Africa. Despite its relatively small sample size, this is the first longitudinal cohort study to describe the socio-demographics, risk factors, treatment, and outcomes of those diagnosed with colorectal cancer in Johannesburg, South Africa (18), hence, a valuable analysis that will hopefully serve as a basis for a broader validation in our setting. We have also validated the predictive models internally using three validation methods. Since there is no available external source test data to validate these models externally, we proposed to externally validate the current predictive models and ascertain the generalizability of these models using the upcoming longitudinal CRC study.

In conclusion, this study demonstrated the ability of LR and SVM to produce a clinically helpful model in predicting patient hospital LOS with high performance. The association established in this study may enable clinicians to implement changes in patient care pathways. If data were collected in the pre-and the post-hospital environment, a broader understanding of poor outcomes would be achieved. Seeing the bigger picture may enable clinicians to depersonalize poor outcomes and focus on measures beyond the failings of individuals. Such a strategy would not only improve clinical outcomes but is also likely to improve efficiency and, therefore, favorably impact the cost of care for patients with CRC. Finally, this study demonstrates that there is so much needed to be done in order to enable the management of CRC in South Africa to be comparable to that of the developed world.

## Data Availability Statement

The raw data supporting the conclusions of this article will be made available by the authors, without undue reservation.

## Ethics Statement

Ethical approval for this study was obtained from the Human Research Ethics Committee (Medical) of the University of the Witwatersrand, Johannesburg, South Africa (M1911131). The patients/participants provided written informed consent to participate in this study. Written informed consent was obtained from the individual(s) for the publication of any potentially identifiable images or data included in this article.

## Author Contributions

OA conceptualized, analyzed the data, compiled the results, and wrote the manuscript. DB and JF provided the data and reviewed the manuscript. ES and GN reviewed the manuscript. ME and EM supervised the concept development and reviewed the manuscript. All authors contributed to the article and approved the submitted version.

## Funding

The CRCSA study was funded by the Medical Research Council of South Africa through the Wits/SAMRC Common Epithelial Cancer Research Centre (CECRC) Grant (Paul Ruff, principal investigator). The DELTAS Africa Initiative supports the present study. The DELTAS Africa Initiative is an independent funding scheme of the African Academy of Sciences’s (AAS) Alliance for Accelerating Excellence in Science in Africa (AESA). The New Partnership supports it for Africa’s Development Planning and Coordinating Agency (NEPAD Agency), with funding from the Welcome Trust [grant 107754/Z/15/Z- DELTAS Africa Sub-Saharan Africa Consortium for Advanced Biostatistics (SSACAB) programmer] and the UK government.

## Conflict of Interest

The authors declare that the research was conducted in the absence of any commercial or financial relationships that could be construed as a potential conflict of interest.

## Publisher’s Note

All claims expressed in this article are solely those of the authors and do not necessarily represent those of their affiliated organizations, or those of the publisher, the editors and the reviewers. Any product that may be evaluated in this article, or claim that may be made by its manufacturer, is not guaranteed or endorsed by the publisher.
